# John Howard's Observations on Hospitals (1773-1790)

**Published:** 1905-11-18

**Authors:** Henry M. Hurd

**Affiliations:** Superintendent of the Johns Hopkins Hospital, Baltimore, Md.


### HOSPITAL ADMINISTRATION.

### CONSTRUCTION AND ECONOMICS.

### JOHN HOWARD'S OBSERVATIONS ON HOSPITALS (1773-1790).

By Henry M. Hurd, M.D., Superintendent of the Johns Hopkins Hospital, Baltimore, Md.

Charitable and humanitarian impulses did not seem to be very active in any nation, or among any people, during the eighteenth century. The slave trade flourished principally through the energy and enterprise of so-called Christian nations, and was regarded a legitimate industry even for religious men. Torture was inflicted in many prisons in Europe as late as the third quarter of the century to extort confessions from persons accused of crime, who were thereupon, often when half dead from the cruel ordeal, promptly executed. Mercenary soldiers were sold to warring nations throughout Europe, and were sent to America that electors and kings might live luxuriously upon the price of flesh and blood thus sold. In Connecticut prisoners were confined underground, without light, air, or comfort, in a deserted copper mine, to save the expense of building a penitentiary. In many States of the American Union the care and maintenance of the friendless and witless were annually awarded to the lowest bidder, and no questions were asked as to the competency of the bidder to give proper care or sufficient food. The whipping-post and pillory were still considered useful, nay indispensable, in dealing with minor offences. England inflicted capital punishment for 160 different crimes, and was reluctant to diminsh the fearful list. This list of inhumanities might be indefinitely extended, but the brief enumeration of these well-known facts will suffice to establish my assertion.

It was not until 1773 that John Howard began his philanthropic work in behalf of prisoners in gaols and workhouses, moved thereto by his personal observations while High Sheriff of Bedfordshire. During the following seventeen years, until his death in Russia in 1790, he travelled throughout England and the principal countries of Europe to inspect and to report upon the condition of prisons, lazarettos, and hospitals. In prisons he found many abuses. To quote from Lecky's *England in the Eighteenth Century*, which contains a summary of his work, he found: "The food in nearly all English prisons was utterly insufficient. The pennyworth, or at most two pennyworths, of bread daily allowed each prisoner had been originally fixed at a time when corn was nearly twice as cheap as when Howard wrote, and being very frequently farmed out by gaolers, the amount was constantly diminished. In nearly half the county gaols, the debtors, and in several bridewells (or houses of correction) all prisoners were left without any regular allowance of food, and subsisted on charity. There were often no sewers, no infirmaries, no means of warming the prisons during the winter. In one gaol Howard found but three pints of water a day allowed to each prisoner for both drinking and washing. Prisoners were crowded to excess for fourteen or fifteen hours of the day in dark, damp, subterranean dungeons reeking with pestilential effluvia. In many gaols and most bridewells there was no allowance for bedding or for straw for prisoners to sleep on, and if by any means they procured any, it was not changed for months. Almost all ventilation was stopped in order to escape the window tax. The vileness of the air was such that Howard declared that after visiting the prisons his clothes were so impregnated that he could not bear to drive in a post-chaise with closed windows, but was obliged to travel on horseback, and even the leaves of his memorandum book were so tainted that he was unable to use it until he had spread it for an hour or two before the fire. In such an atmosphere of putrescence and filth human life rapidly withered. Scorbutic diseases multiplied fiercely; mortification in the feet was so deadly that some great contractors for transporting criminals to the Colonies complained that the mortality from this cause alone almost destroyed their profits; discharged prisoners proved the centres of contagion wherever they went, and the gaol fever raged with such a deadly virulence, Howard computed, that every year it carried away far more than perished by the gallows."

Howard's record of these inspections is contained in two finely printed books, which ran through several editions, and which proved very effective weapons in the warfare which he waged against the manifold abuses which had grown up around the whole prison system. The first work, published in 1777, is entitled *The State of the Prisons in England and Wales, with Preliminary Observations and an Account of some Foreign Prisons and Hospitals*, and the second, published in 1789, a few months before his death, was entitled *An Account of the Principal Lazarettos in Europe with Various Papers relative to the Plague; together with further Observations on some Foreign Prisons and Hospitals and Additional Remarks on the Present State of those in Great Britain and Ireland.* These books have been placed, through the efforts of Miss Nutting, of the Johns Hopkins Hospital, in the library of the Training School for Nurses, and were thus brought especially to my attention. They would be profitable reading for all persons interested in hospital construction and management. Before I read them I supposed that they related to prisons solely, but I found upon a closer examination that they contain equally interesting accounts of hospitals and infirmaries. The works show that Howard had clear ideas of hospital abuses and reforms. There is every reason to think that hospitals, as well as prisons, have profited by his investigations and recommendations.

Howard and Hospital Reform.

Howard's attention seems to have been attracted at first to hospital reform by the condition of the infirmaries which were attached to prisons, gaols, and workhouses, and his views as to their organisation and equipment are very succinctly stated. He believed that an infirmary should have a bath attached to it, so that the sick could be rendered clean and comfortable. He thought that medical attendance and medicine should be provided, also a free allowance of good food, such as milk and wine. He urged the use of soap and a supply of clean linen. Above all, he insisted upon an abundant supply of fresh air, and proper nursing by good nurses. In one instance he spoke of the great advantage of women nurses as more "cleanly and tender." He recommended special precautions against infection, and spoke of fumigation and the propriety of exposing all clothing, which had been infected, to heat in an oven. If this should be impossible, he recommended that they be buried in the soil. He urged that special rooms be provided for convalescents, and insisted that such convalescents should be thus gradually prepared, by suitable apartments and diet, for their final return to a prison diet and hard labour.

*(To be continued.)*

